# Dimensions of social capital of families with thalassemia in an indigenous population in Tamil Nadu, India – a qualitative study

**DOI:** 10.1186/s12939-017-0609-8

**Published:** 2017-06-24

**Authors:** Bharathi Palanisamy, Kalpana Kosalram, Vijayaprasad Gopichandran

**Affiliations:** 1 Doctoral Research Scholar, School of Public Health, SRM University, Kancheepuram District, Tamil Nadu India; 2 School of Public Health, SRM University, Kancheepuram District, Tamil Nadu India; 3 Department of Community Medicine, ESIC Medical College & Postgraduate Institute of Medical Sciences and Research, KK Nagar Chennai, 600078 Tamil Nadu India

**Keywords:** Social capital, Social determinant of health, Thalassemia, Indigenous population

## Abstract

**Background:**

Studies have shown that social capital is positively associated with health, and the association is context-based. Indigenous populations with poor access to health care largely depend on social capital for their health care needs.

This study was conducted to explore the dimensions and types of social capital and its utilization by families with thalassemia for their health and well-being in an indigenous population in Tamil Nadu, India.

**Methods:**

The participants in the study were parents who had children with thalassemia, belonged to an indigenous community in Tamil Nadu, were poor and marginalized, and had poor access to health care. Different dimensions and types of social capital were examined with the help of qualitative in-depth interviews using a phenomenological approach. A total of 8 in-depth interviews were conducted and transcribed. Thematic analysis of the data was performed.

**Results:**

The social capital identified through the in-depth interviews consisted of various levels of family support, financial support from relatives and neighbors, the provision of information from formal and informal networks, and trust in the physician. Indigenous communities are close-knit due to their geographical remoteness and limited accessibility. Family ties were a form of social capital that encouraged bonding, and provided support and care to the children affected by thalassemia. The bonding also helped to meet the regular requirement of blood donation for the children. Relatives and neighbors were an asset that served as a bridge for the families affected, helping them in times of immediate and urgent financial need, making it easier to sustain long-term treatment and providing emotional support. There were informal networks that bridged parents belonging to indigenous and non-indigenous communities, with the latter providing the former with information to help them choose better health care at an affordable cost. The other formal links were the ties between the parents and nongovernmental organizations, such as the local thalassemia association, which connected members belonging to different areas. It was these ties that were of the greatest assistance to the families affected in coping with the disease, enabling them to sustain the treatment, and assisting them to choose and carry out the complicated bone marrow transplantation, which is the definitive treatment for this condition.

**Conclusion:**

The bonding, bridging, and linking dimensions of social capital help communities cope with thalassemia, the more so in indigenous and marginalized communities.

## Background

Social capital refers to networks of social relationships that provide resources and support to individuals and groups within the networks [1]. Social capital is an individual construct and it is the ability of an individual to benefit from the resources possessed by associations and social structures [2]. It is a multidimensional concept that acts both as an individual and community-level determinant of health [3, 4]. Social capital can be perceived of as existing in two forms. In the structural form, it is objective and measures the individual’s actions and behaviors, and in the cognitive form, it is subjective and measures the individual’s attitudes and perceptions [5]. Recent work in public health highlights the distinction between the bonding, bridging, and linking dimensions of social capital [6, 7]. Bonding social capital comprises relations within homogeneous groups, namely, strong intra-group ties that connect family members, neighbors, and close friends. Bridging social capital refers to weak ties that link people from different ethnic, occupational backgrounds. It includes formal and informal social participation. Linking social capital exists among those involved in hierarchical or unequal relations due to differences in power, status, and resources.

Research on association between social capital and health started in the mid-1990s [8]. Wilkinson (1996) introduced the concept of social capital to public health. This was heralded by a greater focus on a society’s economic and social structure as a determinant of health. Important determinants of health include the organization of the society, social interactions among community members, trust between them, and the care given to them [9]. Social capital, which reflects the social processes, norms, networks, and trust that exist among communities, is an important resource for the promotion of health. [10] This is because it helps in the diffusion of health-enabling knowledge, influences healthy behavioral norms, enhances access to health care services and facilities, and gives rise to affective support and mutual respect through psychological processes [3, 11]. It brings contemporary ideas and innovations to people belonging to a marginalized community through their link with power [12]. Participation in social networks provides emotional and instrumental social support, which may be associated with health, through psychosocial, behavioral, and physical pathways [13, 14]. Social interaction also helps to inform people about prevention of disease, and the appropriate health care set-up for preventive and curative services [15]. Thus, social capital has a positive association with health.

Some empirical studies in the developing world have revealed the association between social capital and health. A study from rural Bangladesh showed that self-care for diarrhea, including the use of oral rehydration solution, was successful when an individual and household had access to social capital assets, such as information on health, social support, and resources [16]. A study done in Taiwan described various forms of social capital that encouraged protective behaviors during an influenza epidemic. It showed that the bonding and linking dimensions of social capital enhanced the acceptance of vaccines and hand hygiene, thus reducing the risk of influenza. Wearing face masks was associated with all three forms of social capital [17]. A study on social capital and the utilization of health care in India showed that diverse, heterogeneous social ties were positively associated with utilization of antenatal care, professional birthing care, and childhood immunization [18]. Moreover, social capital may safeguard marginalized communities and protect them from the worst effects of vulnerability by providing them better information, access to resources, and financial, intellectual, and emotional support [19].

Indigenous communities are marginalized due to poor access to resources, lack of education, and poor geographical accessibility. In this context, people’s social assets are limited to their immediate family and communities, the local government, community-based organizations or religious institutions. It is from these assets that people derive essential resources and services. Therefore, studying the existing social resources at the community level and the support provided by the indigenous communities is important. A good understanding of the dimensions and types of social capital among indigenous populations would help find sustainable ways for planning health services, keeping the limitations of resources, access to information and health care in mind.

Hemoglobinopathies are an important national health burden in India. Among other hemoglobinopathies, beta thalassemia is an important health problem that receives little attention because of other competing priorities, such as tackling the massive burden of protein energy malnutrition and communicable diseases. In India, about 45 million thalassemia carriers and 15,000 children are born with full-blown thalassemia each year [20]. Tamil Nadu, a state in south India, has a particularly high burden of thalassemia, with a prevalence of 4% [21, 22]. Thalassemia is a genetic, autosomal recessively transmitted disease. The synthesis of hemoglobin is deficient and hence, children affected by this disease have a severe form of anemia. Children affected by beta thalassemia major need long-term and sustained life-saving treatment, in the form of regular blood transfusion and iron chelation treatment. This makes it imperative for the indigenous families to seek information, resources, and support from within their community, as well as other communities which have access to such care. For these reasons, this disease serves as an important marker to understand the dynamics of social capital as a determinant of health and treatment-seeking behaviors among indigenous communities. It is necessary to understand the perceived social capital among families which have members affected by thalassemia, and also, the extent of information they have, their access to health care and the support derived from social capital. To fulfil this need, we carried out this qualitative study among the families of children affected by thalassemia in an indigenous tribal population in Tamil Nadu.

### Context of the study

This study was conducted in Tamil Nadu, located in south India. As per the decadal census of 2011 in India, the proportion of indigenous tribes in Tamil Nadu was 1.1%. This study focuses on the Malayali tribes settled in one of the hilly areas of the Eastern Ghats (mountains) of Tamil Nadu. This community has several features of indigeneity, such as cross-cousin marriage, which is one of the risk factors for thalassemia [23]. The ‘trial’ marriage system, strong homogeneity of the community, unique rituals and traditional practices, and unique religious rites are the other features of indigeneity in this community. However, many of these cultural characteristics have changed over the past 30 years, in keeping with the large-scale migration of men and women to the plains in search of work [24]. The Malayali tribes live mostly in hilly, geographically inaccessible terrain and their main occupation is agriculture. Very few non-indigenous populations are found in these areas. Despite the apparent homogeneity of the population, there are subtle social and class hierarchies within the tribal community, reflected in sociocultural practices and religious rites. Geographical inaccessibility restricts many opportunities for social attainments among these populations, such as education, economic development, and social support. Remoteness and social norms encourage the members to marry close relatives. As autosomal recessive diseases have a tendency to affect populations with a strong tradition of consanguineous marriage, this area has a high incidence of thalassemia. A segment of the Eastern Ghats, inhabited by an indigenous tribal population, is one of the hotspots for thalassemia in Tamil Nadu. A large-scale population-based study showed the prevalence of thalassemia to be around 1.48–3.64% [25]. A project carried out in this area by a nongovernmental organization (NGO) showed the prevalence of thalassemia trait (mild form of thalassemia) to be 2.2%[26]. The people belonging to this community live in conditions of poverty, the average per capita daily income being about INR 150 (~3 USD). Generally, these indigenous communities have poor access to information on health and hence, their utilization of health services is low. Given its long-term treatment and the sophisticated treatment methods, such as bone marrow transplant, thalassemia imposes a heavy financial burden on the patient’s family. The need to travel long distances to the plains to have the patient treated increases the family’s burden further. In this context, social capital through social relationships within and between communities is the main source of information and support for accessing the health care system. This study is part of a doctoral research of the first author, who is an anthropologist interested in the field of public health, in the broad area of social capital and health. Using thalassemia as the marker disease, the doctoral research attempts to understand social capital and its role in health among indigenous tribal communities. It involves two components: the qualitative study (current study) that explores the dimensions of social capital, and a quantitative follow-up study that attempts to quantify the extent of social capital and its influence on treatment-seeking behaviors among those with thalassemia. The study examines the dimensions and types of social capital available to the indigenous community with respect to access to better treatment and assistance for thalassemia. The main reasons for selecting this disease and this specific population are threefold: (i) its indigeneity, (ii) thalassemia-affected families suffer from a great social and financial burden, (iii) the importance of social cohesion and capital for treatment-seeking behaviors for this condition, especially for indigenous and marginalized communities. Future studies may be required on people suffering from other diseases, and in other non-indigenous and non-poor populations to elaborate the different facets of social capital and health.

## Methods

This study used a qualitative research method. It used a phenomenological approach wherein the focus is to gain an in-depth understanding of the selected population’s lived experiences, perceptions, relationality with family, community, government, and NGOs, and other social ties, all of which have an influence on the experience of living with thalassemia. In-depth interviews were conducted with purposively sampled parents of children affected with thalassemia.

### Ethics statement

The study procedure was discussed and approved by the institutional review board and ethical committee of the institution from which this study originated. As the levels of literacy of the indigenous communities were low, the use of a written informed consent document was not feasible. The parent was interviewed and verbal informed consent was obtained from him/her. A note signed by the researcher conducting the interview was maintained, documenting the process of verbal informed consent. The verbal informed consent process was approved by the ethical committee. The children were present during the interview process; however, they were not asked to assent to having their condition discussed with the researcher. This was because the interview largely focused on the experiences of the parents living with the child. The matter was also discussed in the ethics committee and it was felt that such explicit informed assent by the children was not necessary in the cultural context of the study.

### Sampling

To achieve the main aim of the study, the sample was selected from the indigenous population living in the hilly area described earlier. The families with children with thalassemia were identified on the basis of the first author’s prior experience of working in the community, and with the help of the head of the local governance body,. The families of the affected children were approached and the parents available interviewed. A total of eight families were visited and either the father or mother was interviewed, depending on who was available. Among the parents interviewed, 5 respondents were women and 3 men. Saturation of information in terms of the social capital available for various aspects of handling the illness was achieved in 6 interviews. Two more interviews were conducted to validate the saturation, and the interviews were stopped at the number of 8 since saturation of themes was confirmed., Seven of the affected children among the families interviewed were boys and one was a girl. All the children were between the ages of 5 and 17 years.

### Interviews

In-depth interviews, which are intensive and individual and are held with a small number of respondents, are useful for exploring particular ideas, concepts and personal experiences [27]. Therefore, individual in-depth interviews were preferred for exploring individual ideas and experiences regarding the concept of social capital and how it is useful to overcome particular health problems in a marginalized community. The first author, who conducted all the interviews, practised the methods of bracketing and intuiting to stay true to the phenomenological approach. She ensured that her preconceived ideas (emerging from her prior experience of working in the area) were documented and bracketed off from the interviews. The interview was kept open-ended and there was no structured interview schedule. She also attempted intuiting by arriving at meanings of lived experiences as the interviews proceeded. All the interviews were conducted in the months of September and October 2016. They were conducted at the respondent’s residence and in the vernacular, Tamil. The interviewer initially conversed about the health status of the affected child and then about the family’s perceptions of the community and other support, experiences and availability of health facilities to overcome the child’s health problems. The interviewer explored different aspects of social capital, such as how the families identified the disease, the support provided to and received by their children with respect to the disease, and the utilization of social capital for the health and well-being of their children. The interviews lasted a minimum of 30 min to a maximum of more than two hours. They were audio recorded after obtaining permission. Further, extensive notes were made in the field.

### Coding and analysis

The notes taken during the interviews were elaborated and enriched with audio recorded information. Both the field notes and transcripts, translated into English, were used for analysis. The study followed a phenomenological approach, which emphasizes people’s lived experiences. It helps one to better comprehend the people’s perceptions, perspectives, experiences and understanding of phenomena. Phenomenological enquiries focus on experiences and their interpretations, and do not attempt to explain cause–effect associations or objective reality. Therefore, no theoretical model was required to start the enquiry and none was used. The first researcher read the notes many times and coded them to identify the main themes and ideas emerging from the in-depth interviews. Open coding was performed by identifying the ideas related to the theme. In the second stage, the researcher collected all the phrases and ideas from the initial coding and grouped them into broader themes. A conceptual framework was developed to understand the themes collected. Analysis was performed by only the first researcher. However, the themes were constantly discussed with and agreed upon by the second researcher to ensure a level of analysis triangulation. The third researcher was involved at a later stage to further triangulate the data analysis.

### Reflexivity

The fact that the interviewees were the parents of affected children and shared their feelings and emotions during the interviews evoked strong emotions in the researcher. The researcher’s reactions may have influenced the respondent’s narratives. The researcher maintained memos in the reflection journal, indicating specific instances during the interviews of the possible influence of these emotional states. These memos were considered during the coding and analysis. Reflexivity is the systematic and conscious awareness of the context in which knowledge is generated at every step of the research. Therefore, reflexivity was maintained at the design, interview as well as data analysis stages. The aim of reflexivity is not to do away with the subjectivity of the research, but to be conscious and aware of the biases and subjectivity, and to interpret the findings in view of this context. This was achieved by maintaining a log and constantly reflecting on the subjective biases of the researcher.

## Results

**Fig. 1:**
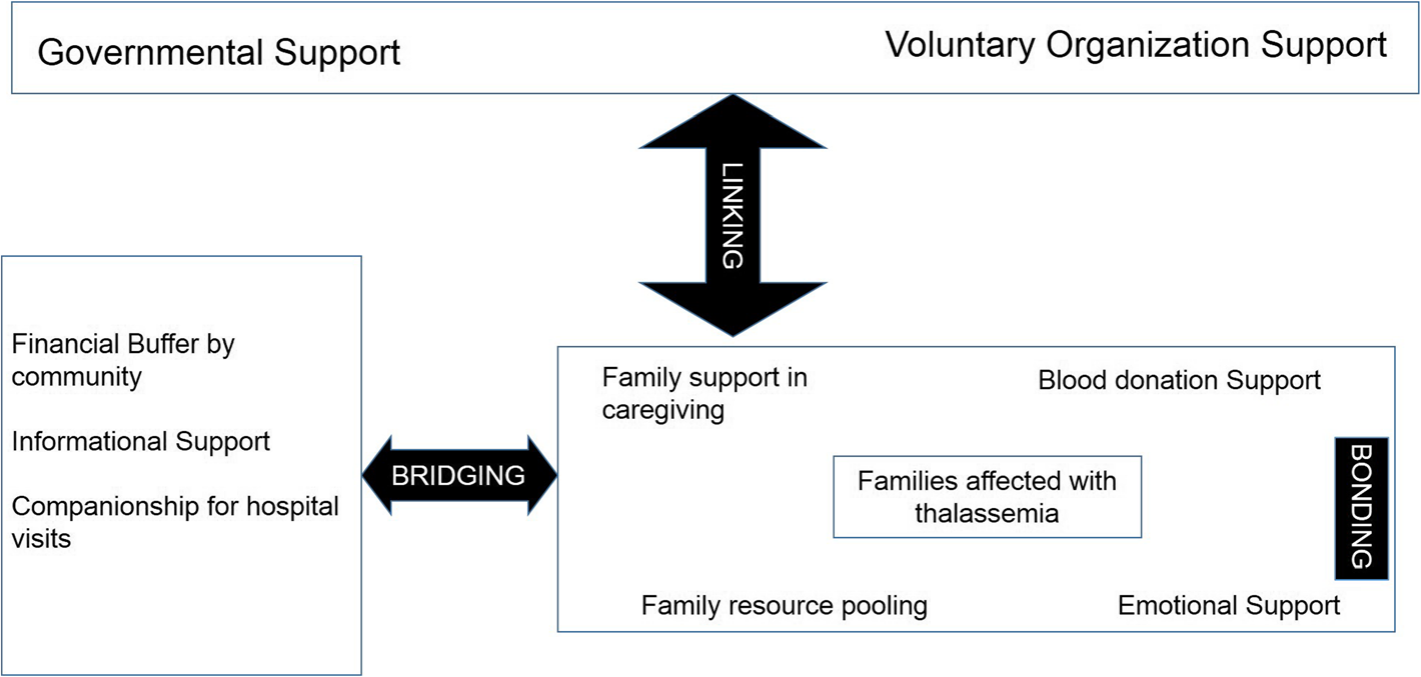
Conceptual framework of social capital among families affected by thalassemia

The main dimensions and types of social capital with regard to families with children with thalassemia are shown in Fig. 1. They are described in detail below.

1. Family support in caregiving
  Family members are best suited to provide effective and sustainable support to persons with long-term illness. Family ties are a strong form of bonding social capital. The family is the prime social unit and the first institution of which the child is a member. It contributes to the development and socialization of children and is the foremost base for the provision of social support [28]. Since thalassemia occurs in early age, the family’s support is imperative to maintain the health and well-being of the affected child. Children affected with thalassemia experience substantial disruption of education and difficulty in carrying out regular social activities. They frequently miss school because of regular hospital appointments for blood transfusion and tackling complications of treatment. When caring for the child is excessively burdensome for the parents, it becomes essential for them to fall back on their families for support. Previous studies on family social capital have found that social capital has a positive impact a child’s health, development, and well-being [29, 30]. Family support in caregiving comes in three important forms – intra-familial division of caregiving responsibilities and job delegation; the provision of support by the extended family when the immediate family is overwhelmed; and the provision of support by the extended family to take care of other unaffected children and the rest of the family when one family member is busy looking after the affected child. In some affected families, both parents were involved in caregiving. However, due to the dearth of job opportunities in the hills, the men usually migrate for work. This placed many women in the position of being the sole caregivers on a day-to-day basis. The mother of an affected 8-year-old boy said:
*Due to the poor livelihood opportunities in the hills and need for money for regular treatment of our boy, my husband has to go out of the village for work. I stay back and take care of the day-to-day activities of the child. This way we manage the family.*
  Thus, cohesion within the family, delegation of responsibility and division of duties became important means of providing family support in caregiving. Caregiving for a child with long-term illness can also be extremely stressful. It is during these situations that the support of the immediate family came in handy. A mother fondly recalled the role of an unaffected child who helped look after her younger brother (8 years old), a thalassemia patient.
*All members in my family take care of him (child affected by thalassemia) and his sister is particularly very supportive in giving care.*
  Grandparents and other family members who lived nearby also helped to take care of children with thalassemia. The mother of an 8-year-old boy affected by thalassemia said:
*Living in deprived conditions and caring for the affected children regularly is difficult without the support of my father-in-law and mother-in-law. Due to lack of employment, the child’s father has to go to work outside the village and earn money for the treatment and the family expenditure. I also go for seasonal coolie work (wage labour). When neither of us is at home, my child’s grandparents take care of him.*
  Sometimes, when the parents are busy looking after the child with thalassemia, the other children in the family are actively supported by their grandparents and other immediate family members. The mother of a 5-year-old boy with thalassemia said:
*My husband used to go to urban areas in search of work and usually I have to take my child to the hospital for treatment. I have a 2-year-old daughter, who also needs to be taken care of. My mother supports me in taking care of her when I have to go to the hospital with my son.*
2. Emotional support by family
  The family also provided the required emotional support when the parents were overwhelmed by the stress of caring for the child with thalassemia. The ability to show empathy, compassion and genuine concern for others is called emotional support. Emotional support is one of the most basic social needs of an individual. Social capital is essential for families in need of emotional support, especially in the case of those affected by a child who is ill. Families act as cohesive bonds in times of stress, and in resource-deprived settings, intra-familial cohesion and emotional support play an important role in helping the members cope with the stress of caregiving. The mother of a 10-year-old boy said:
*My child is alive because of my father, who gave us the emotional support and encouragement to motivate us to go for bone marrow transplantation treatment, and now my son is healthy.*
3. Family resource pooling
  The economic resources of the family play a key role in the continuation of treatment of children affected by thalassemia. The fact that the affected families belonged to a marginalized community and the local health system lacked the facilities to treat thalassemia increased the economic burden on the members further, considering that they had to travel long distances to access care. Due to these circumstances, the affected family had to depend on the pooled financial resources of the extended family. The mother of a 10-year-old boy who had undergone the expensive procedure of bone marrow transplantation reported:
*Because of my father’s financial support, we were able to do bone marrow transplant for my child at a private hospital in Chennai. My father has financially contributed a lot to meet my child’s treatment expenditure, selling agricultural products and through his various contacts.*
  Family support is a multidimensional concept and is directly associated with physical and psychological health [31]. Pooling of resources in the extended family is an important form of social capital [31]. It is seen both as a moral obligation as well as a responsibility of the family members toward one another. Since the family is a cohesive unit, there is also a sense that the pooling of resources is a sound investment for a potential future reciprocal need.
4. Financial buffer provided by neighbors and friends
  Economic disadvantage, a condition of chronic and complex disease, lack of proper health facilities and the high cost associated with regular treatment compound the economic burden of poor families affected by thalassemia. For example, families that have children with thalassemia have to go to Chennai (the capital city, about 300 km from the remote indigenous area) for treatment twice a month, for which a minimum of INR 2000 (~30 USD) is required. Social capital at the individual level serves to reduce stress and increase the ability to overcome it by means of financial and emotional support [32]. The parents of children with thalassemia felt that they received a financial buffer from their relatives and neighbors, which was a source of bridging social capital. The mother of an 8-year-old affected child recalled the financial aid received from her relatives and friends:
*Many times my brother, sister and my husband’s friends helped us financially to enable us to go for blood transfusions and treatment. Sometimes we turn to our neighbors too for financial aid, and then repay it or pay them back by doing agricultural labor on their land.*
  She also said:
*Always we turn to our relatives for our financial needs and have a minimum of 20–30 such relatives in our village.*
  Since most of the relatives of these families lived in close proximity to each other, they were willing to help financially because the proximity gave them a sense of security with respect to the money. However, friends and relatives living in the same community also faced financial hardship in other aspects of their lives, though not in terms of thalassemia, so the extent of their support was limited by poverty. The father of a 7-year-old girl said:
*More than our relatives, I have turned to our neighbors when in financial need. If they have money at that time, they will give it to me. They also go out for manual labor like us and so sometimes, they may not have money to spare.*
  One parent whose 17-year-old son had undergone bone marrow transplant said:
*Apart from financial assistance from the government, the doctor asked us to arrange a minimum of INR 2 lakh (3000 USD) for other expenditure, such as accommodation and food. We had to turn to all our relatives, neighbors and known people for financial assistance. Since most of them were in a similar economic condition as us, we were able to collect only INR 10,000 (150 USD) through these sources.*
  Therefore, though the financial buffer offered by friends and neighbors was perceived to be significant, it did not suffice to compensate for the huge financial burdens imposed by the disease as most of the friends and neighbors faced similar financial hardship.
5. Support for blood donation by family and friends
  Hospitals generally request the families of children with thalassemia to donate blood to replace the blood transfused to the child from the blood bank. In most of the interviews, it was found that it was family members and close relatives who donated blood at the time of need. The motivation to donate blood is a social phenomenon that arises from solidarity within the community [33–35]. Blood relationship, affection and the fact that the children affected were very young motivated the relatives and extended family to donate blood. Previous studies in a developed country have shown that blood donation is positively correlated to social capital and a feeling of belonging to the community. [33] However, there is scant data from developing countries on the role of social capital in blood donation. In this study, blood donation by the family and relatives was a source of bonding.
  The father of a 7-year-old girl said:
*I visited the district hospital for blood transfusion for my child, but due to low availability of blood, I donated my blood two times. Since I was not allowed to donate during all the visits, I approached my relatives sometimes.*
  The mother of a 5-year-old boy said:
*Due to the lack of availability of blood at the district hospital, they asked us to bring blood donors. Beyond my family and relatives, we don’t have much contact with outsiders whom we can request to donate blood. So my husband, brother and brother-in-law all donated their blood at various times of need.*
6. Informational support by neighbors and friends
  Interactions and the relationships between individuals, families, their peer group and social systems give rise to bridging social support [36]. The parents mentioned that they had many friends, relatives and members of the extended family who were also affected by thalassemia, but they did not keep in touch because of geographical inaccessibility. After the families became familiar with a voluntary health organization in Chennai, which was offering transfusion and chelation treatment, they all started visiting the facility and this treatment centre connected them as families. The affected families exchanged information during these visits. The exchange of experiences and feelings among them helped to reduce their distress. The resilient families explained the strategies which could be followed to face the problem. Social ties and closeness gave them the strength to cope with the situation [37].
  The father of a 7-year-old affected girl said:
*As we had planned to go for bone marrow transplant for my daughter, I contacted another family in the village nearby whose son had got the same treatment and asked about the details, such as how many days they stayed in the hospital before and after surgery, how much they spent and what problems the child would face after the treatment. I kept in touch with the other family constantly in person as well as over phone.*
  Limited access to information impelled the affected families to approach various sources to obtain information on treatment that was inexpensive and of a better quality. Many of the patients informed the interviewer that their children were receiving expenditure-free and good-quality treatment from a voluntary organization in Chennai. The father of an affected child communicated the information to the president of the body for local governance and he,, in turn, disseminated the information to all the families affected. This person was the bridging capital for information for the parents of children affected with thalassemia in the community.
  According to the mother of an 8-year-old affected boy:
*After learning of the nature of the disease and realizing that obtaining treatment every month is unaffordable, we were searching for different hospitals that provided treatment at a low cost. In this context, we came to know of the voluntary organization located in Chennai through the head of the local governance body. The person who informed us about the hospital was a parent of an affected child, who was receiving better treatment free of cost at Chennai. This is how my family came to know about the health facility in Chennai and we are now receiving better and satisfactory treatment free of cost from there.*
7. Companionship for hospital visits
  Companionship for travel to hospital for treatment emerged as a major form of bridging social support. The families of children with thalassemia scheduled their visits to the voluntary organization in Chennai in such a way that they could travel together. Two women belonging to different villages, who would otherwise have felt intimidated at the prospect of travelling all the way to the city, went together for the children’s treatment. Other than getting their children treated, this also served to empower them. The father of a 7-year-old girl child said:
*As I am going out for work most of the time, my wife accompanies my child to the hospital. Other families from the village also go to the same hospital for treatment and this helps them to plan and travel together if the dates of the next transfusion coincide.*
  It was evident that families in this informal network were sharing information and clarifying their doubts, and also helping to balance the task of caregiving.
8. Voluntary organization support
  Voluntary health organizations play a vital role in health management and advocacy among indigenous populations. They help to bridge the gap and create low-cost replicable models in health care. Their activities include the provision of community care, treatment and rehabilitation, research, creation of awareness, training and capacity-building. The Thalassemia Welfare Association was established by a physician as an NGO in this indigenous area. It is a formal linking network of affected families that supports and provides various resources related to the treatment of and medication for thalassemia. The parents of the affected children met at this NGO, shared information and participated in various programs organized by the association. They reported that the association was instrumental in increasing their awareness of the disease. According to the father of a 10-year-old boy:
*Earlier we used to go for blood transfusion whenever my child was physically ill, but after hearing from the Thalassemia Welfare Association about the importance of regular and timely transfusion and about the adverse health effects, such as the inability to walk and the failure of organs due to irregular blood transfusion, I started taking him for regular treatment to the voluntary organization’s hospital.*
  Apart from creating awareness of the treatment of the affected child, the association also provides information on the prevention of thalassemia and offers genetic counseling. One of the mothers, who has a 10-year-old son, said:
*My first child is a boy affected by thalassemia and the second is a normal girl. When I conceived the third time, the Thalassemia Welfare Association doctor asked me to check the fetus for thalassemia trait. Apart from counseling, the doctor arranged for genetic testing through the thalassemia society and identified that the fetus had thalassemia trait. After getting to know the result and receiving counseling from the doctor, I aborted the fetus during my second month of pregnancy.*
  The welfare association also created awareness among the affected families of the social entitlements provided by the government. The mother of a child (10 years old) who underwent the bone marrow transplant procedure said:
*Though we know that we can get free treatment for thalassemia using the government-issued smart card (health insurance card), we do not have a clear idea of how to get the free treatment and medicines by using the card. It is the Thalassemia Welfare Association which informed us about getting treatment and the amount covered under the scheme for bone marrow transplantation, and also guided us on the procedure by which one can avail oneself of it.*
  The association also provided financial support and other subsidies for the treatment of the children. This was perceived by the interviewed families as a great source of support.
  Referring to the travel subsidies that the organization provides to parents to take their children to Chennai for treatment, the mother of a 7-year-old girl said:
*For my son, we have to travel for blood transfusion twice a month to Chennai, which is far away from our village. Now, with the support of the welfare association, we are paying INR 40 (less than 1 USD) instead of INR 125 (2 USD), which is the original train fare.*
9. Governmental support
  When the families affected with thalassemia learnt that bone marrow transplantation was a permanent solution for the problem, they were happy. However, the cost of the treatment, which was around INR 1.5 million (21,000 USD), made it seem like an impossible option for them. Then, through the Thalassemia Welfare Association, they heard about the Chief Minister’s Comprehensive Health Insurance Scheme (CMCHIS), which aims to provide quality cashless medical and surgical treatment to poor families in all government and empanelled private hospitals. It encouraged parents to opt for the permanent solution of bone marrow transplantation, which relieves the affected children from lifelong blood transfusion and medication.
  The father of a 17-year-old boy who had undergone bone marrow transplant said:
*On the whole, we spent about INR 1.7 million (22,000 USD) on the surgery, including all our expenditure. It is impossible without the support of the government. The insurance cover for my son under the CMCHIS was INR 1 million (15,000 USD) and the remaining money was raised with the support of the physician and welfare association through donation and our family’s contributions.*
  To alleviate poverty and empower the poor, the Rural Development and Panchyati Raj (local self-governance) department of the Government of Tamil Nadu implemented a microcredit scheme, called the “Pudhu Vaazhvu” (New Life), with the assistance of the World Bank. Under this scheme, financial assistance is provided to differently abled and vulnerable persons to start livelihood activities. Families with children affected by thalassemia can utilize the fund for their needs and repay the amount in the future, without paying interest. The mother of a 5-year-old affected boy said:
*We received INR 12,000 (170 USD) under the “Pudu Vaazhvu” scheme through our local governance body and it helped to overcome the family’s economic burden to some extent, and it was also utilized during hospital visits.*
  Most of the parents interviewed received financial support under the scheme and felt that though the funds had to be repaid in the future, they helped to ease their economic burden.
10. Trust in health care provider
  Having a trustworthy health care provider is in itself a strong form of social capital. The care provided by the doctor in the voluntary organization in Chennai motivated the parents to continue with and sustain the treatment of their children despite several difficulties. The past experience and personal involvement of the health care provider are important in determining the level of trust [38]. It was evident that if a physician was personally involved, displayed care, and administered satisfactory treatment, the affected families felt sufficiently motivated to travel 300 km to access the treatment every month. The mother of a 5-year-old affected boy said:
*In the government health facility which we earlier visited for treatment, they do not give a date for the next visit and if my child got physical problems, such as difficulty walking or vomiting, only then did we used to go for treatment. But here, in the voluntary organization hospital, the physician gives us a date for the next visit and if we do not turn up on the given date, they call and remind us about the transfusion.*
  Throughout the interviews, the study participants expressed positive opinions on the support provided by the physician, as well as that provided by the voluntary organization thus emphasizing the role of these forms of social capital in treatment-seeking.

## Discussion

This study identified various dimensions of bonding, bridging, and linking social capital in the families affected by thalassemia. These were reported by the families from their own lived experiences. The analysis of the in-depth interviews vividly brought out the phenomenon of social capital in the form of intra-familial and close bonding, community-level informal bridging, and formal hierarchical linking.

Bonding social capital is significant for maintaining the health and well-being of children affected by thalassemia. It was evident from the study that intra-familial cohesion and relationships played a vital role in child care, especially caregiving for children affected by thalassemia. It has been reported that when the family is cohesive and caring, children with thalassemia and sickle cell disorder find it easier to adjust and have a positive body image and characteristics [39]. The family is the main source of support for those with chronic disease, providing as it does tangible assistance in the form of daily care, preparing meals and administering medication. The family also provides emotional, financial, and social support [31]. It was evident that the parents perceived of social supports forthcoming from intra-community social relationships as an important buffer against family crises related to thalassemia. Close family members and relatives also served as the bonding social capital for blood donation. Bridging social capital was identified in the form of support from the members of the extended family and community. This study found that the families of the affected children turned to their relatives and neighbors when they were in urgent financial need, and to meet travel expenses, share their emotions, and obtain information. Our study shows that informal networks between the affected families and their communities are of the greatest assistance to the former in coping with the social, physical, and psychological issues related to thalassemia. Many studies show an association between mental health, on the one hand, and dense and strong horizontal bridging networks, on the other [40–43]. The significance of informal social relationships increases in the presence of poverty and limited access to formal services, such as health care [44, 45].

The literature on chronic illness suggests that different kinds of support and preferential support may be best offered by less intimate and more distant contacts [46]. Bridging social capital affords individuals and the community an opportunity to interact with diverse, heterogeneous groups [47]. The bridging dimension of social capital helps parents to receive information regarding access to services and resources. This influences the utilization of health care [13], serves to support marginalized communities that lack sources of power [48], bolsters modern and contemporary ideas, and enhances the people’s awareness of modern health care services [49]. Socioeconomic deprivation and the chronic nature of thalassemia lead parents to look for resources beyond the internal context of the family and community. Such situations made the interviewed families highly dependent on professionals, and voluntary and government organizations for the treatment of and medications for thalassemia.

It was evident from the study that the affected families identified the disease, and received better treatment, through informal and formal relationships with professionals, and voluntary and governmental organizations. Social ties between communities or community members and the representatives of formal institutions, such as health professionals and social workers, are important for leveraging resources, information, and ideas, particularly among marginalized communities [50]. This is the linking social capital for this community.

The study was successful in identifying the parents’ points of view and lived experiences that spoke of the bonding, bridging, and linking social ties among those with children affected by thalassemia. The participants felt that all types of social capital play an important role in coping with thalassemia, obtaining information, and seeking treatment. In the context of the study’s location, where accessibility and resources were restricted, this was a strong determinant of treatment-seeking behavior. The identification of these types of social capital by this community helps to understand, and thus implement, the best methods of care provision for the affected children. Families with members with thalassemia are in need of physical, social and emotional support. It is their own perception of social capital which helps them cope with the stresses of thalassemia. This is suggestive of cognitive social capital, which is emotional and perceptional. This study has identified social capital through a phenomenological enquiry, thus keeping it realistic and cognitive.

This study has some limitations. One of the main limitations is the lack of data triangulation and methodological triangulation. As thalassemia is not a common condition, there were very few people with the condition. Different methods of data collection could have been attempted had there been a greater number of families available for the interviews. The sample did not suffice for methodological triangulation by attempting various methodological approaches. However, rigorous analysis triangulation was helpful in validating the interpretation of the narratives of the families.

Thalassemia is a chronic blood disorder and affects children at an early age – mostly around 4–6 months. It is a serious public health problem in indigenous populations in India and the affected children need sustainable, lifelong treatment, in the form of blood transfusion and iron chelation medication. The chronic nature of the disease affects the family physically, emotionally, and financially. The study made it clear that it was the tangible and intangible resources received through intra- and inter-group social ties that helped the affected families to cope with the situation and maintain the health and well-being of their children.

These findings have policy implications. In the case of chronic conditions like thalassemia, which require long-term, sustained treatment and regular access to treatment facilities, there is a need for optimal utilization of social capital to facilitate access to treatment. In the case of marginalized populations, a larger proportion of which is severely affected by thalassemia, social capital and social support are essential for effective health care delivery. There is a need for families with thalassemia to actively organize themselves into support groups and social groups to better utilize the health services available. This kind of formal social organization can also advocate for the availability of services to the marginalized community in an accessible form. This can be encouraged and facilitated by the public local governance system. It was seen that linking social capital was vital in providing services to the families affected by thalassemia. Therefore, NGOs and voluntary organizations should actively participate in social support systems and the provision of health care services, especially in marginalized areas. A thorough understanding of the phenomenon of social capital in this community can play a major role in informing and shaping the social security policies, and in planning for better care for thalassemia.

## Conclusion

This qualitative study identified the dimensions of social capital that can be utilized by indigenous families which have children with thalassemia, from their own viewpoint. It was evident from the study that the resources that become available through the bonding, bridging, and linking dimensions served to help the indigenous community to improve their children’s health. Understanding the dimensions of social capital in various communities, from the community’s own perspective, would be useful for planning and implementing effective health care services, especially in resource-poor and marginalized settings.

## Abbreviations

NGO: Nongovernmental organization
